# Viral Infection: A Potent Barrier to Transplantation Tolerance

**DOI:** 10.1155/2008/742810

**Published:** 2008-09-14

**Authors:** David M. Miller, Thomas B. Thornley, Dale L. Greiner, Aldo A. Rossini

**Affiliations:** ^1^Division of Diabetes, Department of Medicine, University of Massachusetts Medical School, Worcester, MA 01655, USA; ^2^Program in Molecular Medicine, University of Massachusetts Medical School, Worcester, MA 01655, USA

## Abstract

Transplantation of allogeneic organs has proven to be an effective therapeutic for a large variety of disease states, but the chronic immunosuppression that is required for organ allograft survival increases the risk for infection and neoplasia and has direct organ toxicity. The establishment of transplantation tolerance, which obviates the need for chronic immunosuppression, is the ultimate goal in the field of transplantation. Many experimental approaches have been developed in animal models that permit long-term allograft survival in the absence of chronic immunosuppression. These approaches function by inducing peripheral or central tolerance to the allograft. Emerging as some of the most promising approaches for the induction of tolerance are protocols based on costimulation blockade. However, as these protocols move into the clinic, there is recognition that little is known as to their safety and efficacy when confronted with environmental perturbants such as virus infection. In animal models, it has been reported that virus infection can prevent the induction of tolerance by costimulation blockade and, in at least one experimental protocol, can lead to significant morbidity and mortality. In this review, we discuss how viruses modulate the induction and maintenance of transplantation tolerance.

## 1. INTRODUCTION

Organ transplantation in the clinic became a reality in 1954 when Merrill, Murray, and Harrison performed the first successful human vascular organ graft, a kidney transplant [1, 2]. However, the donor and recipient were monozygotic twins, obviating the need for immunosuppression for organ graft survival. With the development of immunosuppressive regimens, the same group 5 years later performed the first kidney allograft transplantation between unrelated individuals; that graft survived for 20 years [3]. Although successful graft survival was achieved, it rapidly became clear that all immunosuppressive drugs, even the newer generations of immunosuppressive regimens, are toxic [4, 5]. Immunosuppressive drugs are also known to increase the risk of infection and neoplasia [6, 7], and their associated side effects often lead to patient noncompliance [8]. Since most patients eventually reject transplanted allografts either acutely or through a process of chronic rejection [9–11], these deleterious side effects make organ transplantation a therapy in which the risk/benefit ratio must be carefully weighed.

 To overcome issues associated with chronic immunosuppression, investigators have focused on approaches that lead to the induction of tolerance to transplanted organ allografts [12]. Operationally, transplantation tolerance is defined as the survival of a donor allogeneic graft in the absence of immunosuppression. Most transplantation tolerance induction protocols take advantage of information resulting from studies on the natural mechanisms by which the immune system prevents self-reactivity and autoimmune disease. Two major forms of natural tolerance have been identified: central tolerance and peripheral tolerance.

## 2. CENTRAL TRANSPLANTATION TOLERANCE

In 1953, Peter Medawar et al. obtained the first experimental evidence that the establishment of allogeneic hematopoietic chimerism leads to the induction of central tolerance and permits permanent acceptance of skin allografts [13]. Inspired by the work done in freemartin cattle by Owen in 1945 [14] and the clonal selection theory subsequently proposed by Burnet and Fenner [15], Medawar demonstrated in mice that the transfer of allogeneic hematopoietic cells in utero could induce tolerance to skin transplanted from the original donor later in life [13].

 Medawar's observation led Main and Prehn to experimentally induce hematopoietic chimerism by treating mice with whole-body irradiation (WBI) and allogeneic bone marrow cells, followed by transplantation with donor-strain-matched skin allografts [16]. This protocol successfully induced tolerance to skin allografts, conclusively linking the establishment of hematopoietic chimerism with subsequent allograft survival. However, despite the long-term survival of skin allografts on mice treated with WBI and allogeneic bone marrow, animals eventually develop lethal graft-versus-host disease (GVHD), a reaction where passenger leukocytes in the donor bone marrow or graft mount an immune response against the host. Therefore, modern conditioning protocols to induce central tolerance have been designed to address the common objectives of (1) establishing hematopoietic chimerism using a relatively benign preconditioning protocol that (2) prevents the development of GVHD.

 Despite these common objectives, modern conditioning regimens can differ quite significantly in their methodology. In preclinical models of hematopoietic chimerism, conditioning regimens span the spectrum from myeloablative protocols, which often entail lethal irradiation and subsequent stem cell rescue, to noncytoreductive treatments that do not require irradiation, for example, costimulation blockade [17–19]. Between these two extremes are protocols that significantly weaken the recipient's immune system through selective antibody-mediated elimination of specific immune populations (e.g., CD4^+^ and CD8^+^ T cells) coupled with targeted irradiation (e.g., thymic irradiation) [20]. These latter protocols are often considered nonmyeloablative. In clinical trials, successful nonmyeloablative approaches have recently been described [21, 22]. Stable renal allograft function in recipients for as long as five years after complete withdrawal of immunosuppressive drugs was observed in recipients in which hematopoietic chimerism was established [21, 22]. These reports document that in humans, as in rodents, establishment of hematopoietic chimerism is a robust approach for the development of central tolerance and the permanent survival of donor-specific allografts.

## 3. PERIPHERAL TRANSPLANTATION TOLERANCE

**Figure 1:**
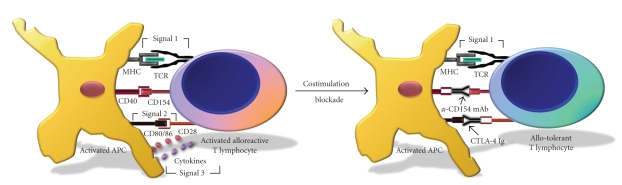
*Costimulation blockade*. Activation of a T cell involves a series of interactive steps with an APC. The first signal imparts antigen specificity and commences when the TCR engages the antigen/MHC complex presented by the APC. This signal is commonly referred to as “signal 1.” In subsequent steps, the T cell receives a number of costimulatory signals, including those following interaction of CD154 on T cells with CD40 on APCs, which matures the APC to upregulate expression of CD80/86. The interaction of CD28 with CD80/86 is termed as “signal 2” and activates APCs to secrete cytokines, which provide the final activation signals to the T cell; this step is commonly referred to as “signal 3.” Protocols based on costimulation blockade can prevent T cell activation by targeting steps in the T cell activation cascade. Anti-CD154 mAb blocks the interaction between CD154 and CD40, and prevents the APC from upregulating CD80/86, blocking full APC activation. This prevents the secretion of proinflammatory cytokines, thus depriving the T cell of signal 3. As a result of costimulation blockade, the T cell does not develop an activated phenotype, and consequently becomes nonresponsive (tolerant) to allogeneic antigens.

The second major form of tolerance is peripheral tolerance. Different from central tolerance in which hematopoietic chimerism leads to the clonal deletion of antigen-specific cells during development, peripheral tolerance targets pre-existing cells that have already been generated. To induce tolerance in this population, fundamental insights into how naive antigen-specific T cells become activated have led to protocols designed to prevent this process. Naive T cell activation is initiated by the interaction of the antigen-specific T cell receptor (TCR) with a peptide presented by the MHC. This interaction conveys specificity leading to the activation of only antigen-specific T cells. This signal is often termed as “signal 1” (Figure 1). Following TCR-peptide/MHC ligation, a T cell then receives a number of costimulatory signals [23–25]. A key costimulatory signal in this pathway that permits the activated naive T cells to become functional effector/memory T cells is provided by CD28-CD80/86 interaction [26], which has often been referred to as “signal 2.” In early studies, it was shown in vitro that T cells that receive signals through their TCR in the absence of engagement of the CD28-CD80/86 costimulation pathway became nonresponsive, a state of T cell nonresponsiveness often referred to as anergy [12, 27]. Following induction of signal 2, cytokines are produced that impart the final signal for T cell activation, and this is termed as “signal 3” [24, 28, 29]. Although these three critical signals are required for the full activation of T cells, additional signals such as those derived from CD40-CD154 interaction can have potent effects on the activation of naive T cells (Figure 1).

 The existence of a comparable in vivo state of T cell nonresponsiveness has been debated for years until it was independently shown to exist by Ohashi et al. [30] and Oldstone et al. [31] using two very similar experimental systems. These investigators generated double-transgenic mice that expressed (1) lymphocytic choriomeningitis virus (LCMV) glycoprotein (GP) [30] or nucleoprotein (NP) [31] under the control of the rat insulin promoter, and (2) a transgenic TCR that recognizes a peptide from the transgenic LCMV protein. In unmanipulated mice, the transgenic T cells migrate from the thymus into the peripheral tissues and encounter their cognate antigen, but they remain nonresponsive to islets expressing GP or NP. However, LCMV infection reverses this state of nonresponsiveness, leading to a diabetic phenotype resulting from the destruction of pancreatic islets expressing the transgenic protein [30, 31]. These data support a mechanism where the LCMV-reactive T cells in naive mice encounter antigen in the absence of costimulation and become nonresponsive (tolerant), and further show that environmental perturbation can break this nonresponsive state. This model serves as the conceptual basis for the induction of peripheral transplantation tolerance, where the in vivo disruption of the costimulatory process—referred to as costimulation blockade—leads to the induction of tolerance in an antigen-specific manner [12].

Costimulation blockade therapies can target several different steps in the process of T cell activation. However, the CD40-CD154 pathway linking signal 1 to signal 2 has been identified to be a critical step in the activation of naive T cells. Anti-CD154 mAb blocks the interaction between CD154 on the T cell and CD40 on the APC [32, 33], and prevents the differentiation between naive T cells and effector/memory T cells [33] (Figure 1).

 In peripheral tolerance induction protocols, anti-CD154 monotherapy significantly improves islet [34] and cardiac [35] allograft survival in mice and islet allograft survival in nonhuman primates [36–39]. In combination with a donor-specific transfusion (DST), anti-CD154 monoclonal antibody (mAb) induces permanent islet [34] and prolonged skin [40] allograft survival in mice. DST provides selective activation of the alloantigen-specific T cells, and we have shown that the subsequent blockade of costimulation by anti-CD154 mAb leads to selective depletion of only the specific alloantigen-reactive CD8^+^ T cells [41, 42]. Another reagent, CTLA4-Ig, binds to the costimulatory molecules CD80/86 on the APC. This blocks its interaction with CD28 on the T cell, preventing signal 2. CTLA4-Ig monotherapy induces the survival of xenogeneic islets [43] and allogeneic cardiac grafts [44]. Not surprisingly, the combination of anti-CD154 mAb and CTLA4-Ig has shown great potential in prolonging skin and cardiac allograft survival in mice [45].

 Effective as a peripheral tolerance induction protocol, costimulation blockade protocols based on blockade of the CD40-CD154 pathway have also been used to establish hematopoietic chimerism leading to the generation of central tolerance [17–19]. By establishing multilineage hematopoietic chimerism, these noncytoreductive protocols have proven to promote robust transplantation tolerance to a variety of solid-organ allografts across fully allogeneic barriers when transplanted several weeks after bone marrow transplantation (BMT) [17, 18] or being concurrent with BMT [19, 46]. Furthermore, because donor-reactivity against the host is dependent on the CD40-CD154 pathway [47], costimulation blockade effectively establishes hematopoietic chimerism in the absence of GVHD [17, 18].

## 4. VIRUS INFECTION AND TRANSPLANTATION TOLERANCE

 As costimulation blockade protocols move closer to clinical reality, there is concern that virus infection during tolerance induction may (1) induce tolerance to the virus, (2) prevent the induction or maintenance of tolerance to the organ allograft, or (3) increase risk to the host. Viruses are known to stimulate innate immunity by activating various pattern recognition receptors (PRRs), such as Toll-like receptors (TLRs) and retinoic acid inducible gene-I- (RIG-I-) like receptors (RLRs) [48]. Activation of innate immunity by virus infection leads to the modulation of adaptive immunity, and it has been shown to impair transplantation tolerance induction and allograft survival [49–57].

For example, infection with LCMV before [54], at the time of [51, 56], or shortly after costimulation blockade for the induction of peripheral tolerance [57] impairs allograft survival. Mice treated with costimulation blockade rapidly reject skin allografts if they are infected with LCMV shortly after skin transplantation [57]. Interestingly, this effect appeared to be virus-specific, as infection with vaccinia virus (VV) and murine cytomegalovirus (MCMV) did not engender allograft rejection [57]. Furthermore, skin allograft survival is significantly shortened in LCMV-immune mice treated with a peripheral tolerance induction protocol consisting of DST and anti-CD154 mAb combination therapies [54]. Additionally, TLRs and their proinflammatory role in responding to infection and ischemia are being increasingly seen as a serious obstacle to solid-organ transplantation [58–60].

Barriers to the induction of hematopoietic chimerism and establishment of central tolerance in the setting of viral infection have also been reported. Anti-CD154 mAb, CTLA4-Ig, and busulfan treatment fails to induce bone marrow chimerism and tolerance to skin allografts in the setting of multiple viral infections [53]. Moreover, using a nonmyeloablative protocol where anti-CD154 mAb treatment was coupled with sublethal irradiation, Forman et al. observed that infection with LCMV on the day of BM transplantation not only resulted in allograft rejection but also proved lethal to the recipient [55]. Interestingly, conditioned recipients that were infected and given syngeneic BM grafts did not die. Recipients of allogeneic BM died by a type I interferon- (IFN-) dependent mechanism, whereas mice deficient in the type I IFN receptor survived. The recent deaths of a cluster of human transplant recipients of LCMV-infected organs make this finding particularly relevant to the safety and efficacy of tolerance induction protocols based on costimulation blockade [61, 62].

## 5. INNATE IMMUNE ACTIVATION BY VIRUS INFECTION

It has been shown that mice infected with LCMV concurrent to costimulation blockade treatment [56, 63] or persistently infected with LCMV clone 13 prior to costimulation blockade treatment [52] rapidly reject skin allografts. In a transgenic TCR model, LCMV prevents the deletion of alloreactive CD8^+^ T cells that is ordinarily induced by costimulation blockade [56, 63]. In this same model system, injection of a TLR agonist similarly prevents the deletion of host alloreactive CD8^+^ T cells which are required for skin allograft rejection [64].

Surprisingly, the TLR4 agonist LPS impairs CD8^+^ T cell deletion and shortens skin allograft survival by activating host cells [64] rather than donor cells [64, 65], even though the transgenic CD8 T cells recognize donor antigen via the direct pathway. Furthermore, LPS required the expression of the adaptor molecule myeloid differentiation primary response gene-88 (MyD88) on the recipient to shorten allograft survival [65, 66]. These findings are consistent with clinical data suggesting that TLR4 polymorphisms on the host, but not the donor, correlate with allograft survival [67]. Together, these data suggest that TLR activation induces a soluble mediator that augments host T cell activation, perhaps through a process of bystander activation (see below).

Numerous cytokines are reported to be important in the activation of CD8^+^ T cells, including IL-12 [29], TNF*α* [68, 69], and IFN-*α*/*β* [70]. While IL-12 and TNF*α* are dispensable for shortened allograft survival induced by LPS in costimulation blockade treatment protocols [64], IFN-*α*/*β* has been reported to be absolutely essential for LPS to prime CTLs and induce allograft rejection [65]. Type I IFNs also proved indispensable for allograft rejection mediated by the dsRNA mimetic and TLR3 agonist poly I:C [65]. Emerging data suggest that IFN-*α*/*β* can be induced by viruses through a growing number of pathogen recognition receptor systems [71–74]. Thus the induction of IFN-*α*/*β* by virus infection or TLR ligation has emerged as an important obstacle to the establishment of peripheral transplantation tolerance as well as to the maintenance of self-tolerance [75].

## 6. SIGNALING PATHWAYS INVOLVED IN INNATE IMMUNE CELL ACTIVATION BY VIRUS INFECTION

**Figure 2:**
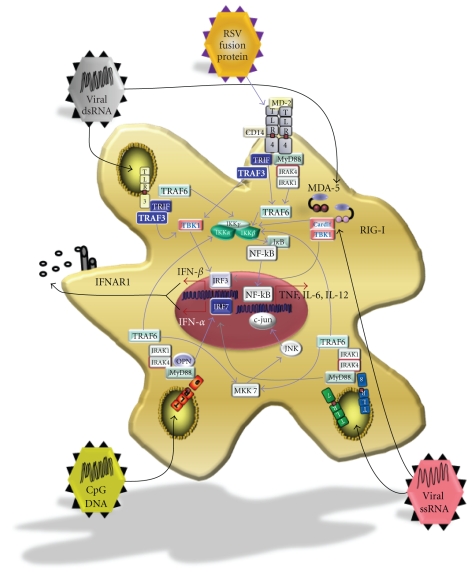
*Pathogen recognition systems*. The innate immune system senses viral pathogens by recognizing distinct pathogen-associated molecular patterns (PAMPs) using various pattern recognition receptors (PRRs). Two of the best-characterized virus-sensing PRRs include member of the Toll-like receptors (TLRs) and retinoic acid inducible gene-I- (RIG-I-) like receptors (RLRs) families. These PRRs couple the recognition of viral PAMPs to the induction of proinflammatory cytokines through various signaling cascades. The cytosolic RNA helicase receptors MDA5 and RIG-I initiate the cascade by recruiting the Cardif/TBK1 complex after sensing viral RNA. This activates the kinase TBK1 to phosphorylate interferon regulatory factor (IRF)-3 and IRF7, resulting in their nuclear translocation and the transcription of IFN*α*/*β*. The cell surface receptor TLR4, in partnership with CD14, couples the recognition of respiratory syncytial virus fusion protein [78] to cytokine induction by signaling through the MyD88-dependent as well as the MyD88-independent pathways. The endosomal TLRs, TLR7, TLR8, and TLR9 also signal through MyD88 to activate inflammatory cytokines such as TNF, IL-6, and IFN-*α*/*β*. The other endosomal TLR (TLR3) signals through the MyD88-independent pathway via the TIR domain-containing adaptor molecule TRIF. Via TRIF, TLR3 signaling can activate NF-kB using TRAF6, and in addition, can induce type I IFN expression probably via TRAF3, TBK1, and IRF3.

 How does virus-mediated activation of innate immunity lead to the production of IFN-*α*/*β*? At present, the two best-characterized IFN-*α*/*β*-inducing viral recognition systems are members of the TLR and the retinoic acid inducible gene-I- (RIG-I-) like receptor (RLR) families (Figure 2). These receptors are activated by sensing viral nucleic acids either in the cytosol (RLR) or in endosomes (TLR) of cells [76]. Cytosolic receptors that detect nucleic acids upon viral infection are expressed ubiquitously by nucleated cells, while endosomal receptors, which detect viral particles that are engulfed from outside rather than from direct infection, are expressed in specialized cells of the innate immune system such as macrophages and dendritic cells [77].

Cytosolic RLRs, exemplified by the proteins RIG-I and melanoma differentiation factor-5 (MDA5), recognize double stranded RNA (dsRNA) located in the cytosol following replication by an RNA virus, or infection by a dsRNA-genome virus, through interaction with their helicase domains [48]. RLRs contain a caspase recruitment domain (CARD) [72] which links detection of viral dsRNA to transcription of IFN-*α*/*β* by forming homotypic interactions with the CARD-containing molecule interferon-*β* promoter stimulator (IPS-1, also known as mitochondrial antiviral signaling protein (MAVS), CARD adaptor inducing IFN-B (CARDIF), and virus-induced signaling adaptor (VISA)) [79–82]. Activation of IPS-1 triggers members of the I*κ*B kinase (IKK) family, specifically TANK-binding kinase 1 (TBK-1) and IKK*ε* (also known as inducible I*κ*B kinase, IKK-i), to phosphorylate and activate interferon regulatory factory (IRF)-3 and/or IRF7 [83–88]. Once activated, IRF3 and IRF7 translocate to the nucleus and bind to interferon-stimulated response elements (ISREs) to induce the expression of IFN-*α* and IFN-*β*, as well as other IFN-inducible genes [48, 89, 90].

 It has recently been recognized that cytoplasmic sensing of DNA can also trigger IFN-*α* and IFN-*β* production [91–93]. This pathway is thought to intersect with the RIG-I and MDA5 pathways at the level of TBK-1 and IKK-I [91], and it requires IRF3 for IFN-*α*/*β* induction [92]. A candidate cytosolic recognition receptor that senses and is activated by DNA has been described [94]. This receptor, known as DNA-dependent activator of IFN-regulatory factors (DAI), was reported to induce type I IFN upon recognition of bacterial and mammalian as well as viral DNAs [94].

With the exception of TLR4, all known TLRs that induce type I IFN recognize nucleic acids, and are found in the endosomal compartment of cells. These include TLR3, TLR7, TLR8, and TLR9. Unlike the cytoplasmic nucleic acid receptors, the cellular distribution of endosomal TLRs is much more restricted. TLR7 and TLR9, which recognize ssRNA [95, 96] and unmethylated DNA that contain CpG motifs [97], respectively, are expressed highly on both conventional (cDC) and plasmacytoid (pDC) dendritic cells. However, they can also be expressed on other hematopoietic cells, including B cells [98, 99]. TLR3, which recognizes dsRNA [71], has a broader distribution than TLR7 and TLR9, and can be found on nonhematopoietic cells such as astrocytes and epithelial cells of the cervix, airway, uterus, vagina, intestine, and cornea [76, 98–100]. Its expression, however, is thought to be highest in cDCs [76, 100].

Similar to the other non-IFN-*α*/*β*-inducing TLRs, TLR3, 7, 8, and 9 are capable of activating both NF-*κ*B and MAPK cascades and triggering the transcription of scores of proinflammatory cytokines and chemokines [76, 99, 100]. However, the endosomal TLRs are also capable of signaling through additional cascades, which results in the expression of type I IFNs. Recognition of dsRNA by TLR3 results in the activation of the adaptor molecule Toll/interleukin-1 receptor (TIR) domain-containing adaptor protein inducing IFN-*β* (TRIF) [101]. TRIF interacts with tumor necrosis factor receptor-associated factor (TRAF)-3 to activate TBK1 [88] and, as described above, leads to the activation of IRF3 and IRF7 and induction of type I IFN. In contrast, the coupling of TLR7 and TLR9 to IFN-*α*/*β* production involves the adaptor molecule MyD88 [97, 102]. Following recognition of either ssRNA or unmethylated DNA, a large complex consisting of MyD88, TRAF3, TRAF6, IL-1 receptor-associated kinase (IRAK)-4, IRAK-1, IKK-*α*, and IRF-7 is recruited to the TLR [48, 87, 88, 103–105]. Following recruitment of the complex, cytokines downstream of NF-*κ*B are stimulated, and type I IFN expression is induced in an osteopontin (OPN) [106] and IRF7-dependent fashion [48, 89]. Interestingly, stimulation of TLR7 and TLR9 in cDCs is capable of inducing the expression of cytokines that are downstream of the NF-*κ*B pathway, such as IL-6 and IL-12. However, only pDCs are capable of producing IFN-*α* in response to ssRNA and CpG-containing DNA [76]. As exemplified by the multitude of signaling pathways by which TLRs can activate innate immunity, it is clear that virus or microbial infection has multiple ways to active innate immunity and modulate the adaptive immune system during tolerance induction.

## 7. MECHANISMS OF VIRUS-MEDIATED MODULATION OF TRANSPLANTATION TOLERANCE

There are multiple mechanisms by which virus infection or TLR agonists may modulate tolerance induction and allograft survival. We will focus on three potential mechanisms. First, virus infection can mature APCs to prime non-cross-reactive T cells, a process called bystander activation [107, 108]. Second, virus infection may stimulate innate immune cells to produce cytokines that suppress tolerance-promoting regulatory T cells [109]. Third, virus infection may lead to the generation of virus-specific T cells that can cross-react with alloantigens, a phenomenon known as heterologous immunity [110].

### 7.1. Bystander activation

 A mechanism by which virus infection may modulate tolerance induction is through bystander activation. As described above, virus infection activates innate immunity, and is able to mature APCs to “license” them to activate non-cross-reactive T cells. CD4^+^ T cells are known to play a pivotal role in the licensing of antigen-presenting cells (APCs) [111]. The intercourse between antigen-specific CD4^+^ T cells and antigen-presenting APCs is thought to be crucial for the generation of a full immune response. In the setting of viral infection, virus-specific CD4^+^ T cells facilitate the maturation of virus-presenting APCs via CD154-CD40 interactions. Consequently, the APC is stimulated to upregulate costimulatory molecules, as well as to secrete proinflammatory cytokines. These molecules then feed back on the T cell, stimulate it to become fully activated, and release additional inflammatory cytokines and growth factors. Allospecific T cells that have encountered cognate alloantigen can be activated in this inflammatory milieu even if they do not cross-react with viral antigens. This process is traditionally referred to as bystander activation [111].

**Figure 3:**
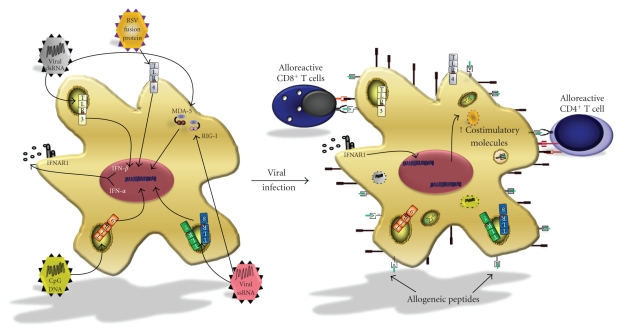
*Bystander activation of alloreactive T cells by “virus-licensed” APCs*. Upon viral infection, detection of pathogen-associated molecular patterns by PRRs can stimulate the production of inflammatory cytokines such as IFN-*α*/*β*, TNF-*α*, and IL-6. These cytokines activate alloantigen-processing APCs in a paracrine or autocrine fashion to upregulate MHC classes I and II, as well as costimulatory molecules, such as CD80 and CD86. The heightened expression of costimulatory molecules elicits the proliferation and differentiation of alloreactive T cells.

Viruses have also been shown to mature APCs independently of the normally required CD154-CD40 interaction. LCMV infection stimulates the upregulation of MHC classes I and II, CD40, CD80, and CD86 in the presence of CTLA-4-Ig and anti-CD154 mAb [51]. The molecular mechanisms that govern this process have not been fully elucidated; however, the induction of type I IFNs by virus-infected APCs is a likely candidate. IFN-*α*/*β* is known to directly induce the maturation of immature DCs, and it results in the upregulation of MHC and costimulatory molecules [112, 113]. Given that pDCs can produce up to a thousand-fold more type I IFN than other cells [113, 114], we propose that viral detection by pDCs triggers the release of IFN-*α*/*β* that can in turn act in a paracrine or autocrine fashion to mature alloantigen-presenting APCs (Figure 3). Thus, these “IFN-*α*/*β*-licensed” alloantigen-presenting APCs could directly stimulate alloreactive T cells, even in the presence of costimulation blockade.

### 7.2. Regulatory cell suppression

The induction and maintenance of CD4^+^ regulatory T cells (Tregs) are essential to allograft survival [115–117]. Therefore, a second mechanism by which viruses could impair tolerance induction is through modulation of the generation or activity of this important T cell subset. In addition to priming cells through an IFN-*α*/*β*-dependent mechanism, TLR activation also prevents the intragraft recruitment of regulatory T cells in an MyD88-dependent manner [66]. This observation extended earlier work showing that the MyD88 pathway plays an important role in the rejection of minor antigens [118] and cardiac allografts [119].

**Figure 4:**
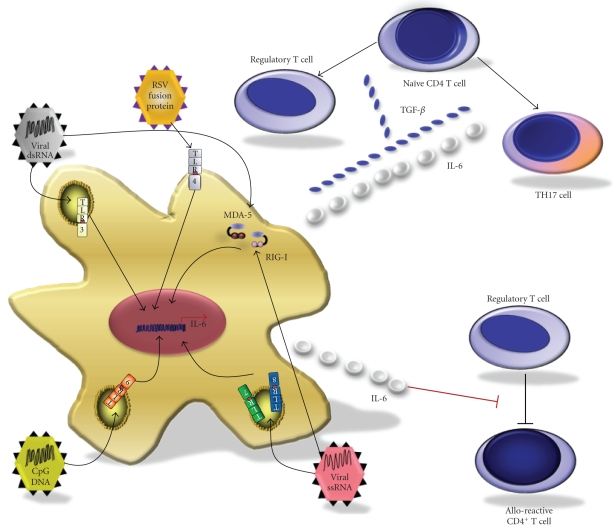
*Modulation of regulatory mechanisms by virus infection*. Regulatory T cells play a crucial role in transplantation tolerance to allogeneic organs. Regulatory mechanisms that prevent immune attack on allogeneic tissues may be compromised in the setting of viral infection by at least two mechanisms. Release of inflammatory cytokines by virus-infected cells can prevent the differentiation of uncommitted naive CD4^+^ T cells into Tregs. Naive CD4^+^ T cells can differentiate into regulatory T cells in the presence of TGF-*β*. However, in the presence of TGF-*β* and proinflammatory cytokines such as IL-6, and perhaps IL-21, naive T cells can be skewed to turn into effector T cells such as the IL-17-producing TH17 cells. In a separate mechanism, release of cytokines such as IL-6 by infected APCs can render alloreactive effector cells refractory to suppression by regulatory T cells.

IL-6 is an MyD88-dependent cytokine that has emerged as a candidate mediator for impairing regulatory T cell generation and function; its production is diminished in untreated [119]—as well as LCMV-infected [120]—mice deficient in MyD88. CD4^+^ T cells develop a FoxP3^+^ regulatory T cell phenotype when they are activated in the presence of TGF-*β*. However, when CD4^+^ T cells are activated in the presence of TGF-*β* and IL-6, this regulatory phenotype is suppressed and the cells develop a proinflammatory TH17 cell phenotype [121] (Figure 4). Therefore, virus infection may precipitate allograft rejection by preventing the generation of Tregs following costimulation blockade and instead favor development of proinflammatory effector T cells.

IL-6 has also been shown to be important in regulating antigen-specific adaptive immune responses via additional mechanisms. Pasare et al. demonstrated that microbial induction of the TLR pathway on DCs enabled effector T cells to overcome suppression by CD4^+^CD25^+^ regulatory cells [122] (Figure 4). They reported that secretion of soluble mediators (principally IL-6) by TLR-activated DCs could render effector T cells refractory to Treg-mediated regulation, permitting activation of antigen-specific T cells in the presence of regulatory T cells. Hence, virus infection may trigger allograft rejection by compromising key regulatory mechanisms such as preventing the generation of regulatory T cells by costimulation blockade as well as by enabling alloreactive T cells to escape Treg-mediated suppression.

### 7.3. Heterologous immunity

The classic view of clonal T cell activation is that one TCR interacts with one cognate antigen. However, we now understand that TCR binding is degenerate, and can recognize multiple related and unrelated antigens. The ability of an antigen-specific T cell to cross-react with multiple antigens, known as heterologous immunity [110], can influence immunodominance, protective immunity, and immunopathology during subsequent viral infections [110, 123, 124].

**Figure 5:**
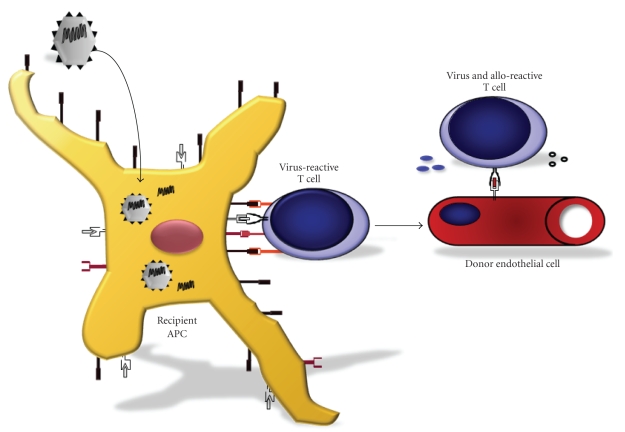
*Heterologous immunity; cross-reactivity between viral and allogeneic antigens.* Unlike the very small proportion of naive T cells that can respond to any given pathogen (reported to be ~1:200 000), the frequency of T cells that directly recognize allogeneic antigens, such as MHC, is thought to be within 1:100–1:10. A proportion of those TCRs that recognize alloantigens, therefore, may have arisen as a result of virus infection that induces virus-specific T cells that cross-react with allo-MHC. Activation of these T cells may result in the recognition of MHC molecules found on donor tissues, such as the endothelium of transplanted organs, precipitating allograft rejection.

In studies of peripheral tolerance induction, of particular interest to transplant scientists is the observation that virus-specific T cells cross-react with alloantigens (Figure 5) [125, 126]. Yang et al. have reported that acute infection with VV, MCMV, or arena viruses LCMV and pichinde virus (PV) resulted in the spontaneous generation of cytotoxic lymphocytes (CTLs) with cytolytic activity towards allogeneic cells [127, 128]. These results were further supported by Nahill and Welsh [126], who used limiting dilution analyses to demonstrate that T cell clones specific for virus-infected syngeneic cells also kill uninfected allogeneic targets. Our report using virus-specific tetramers and an intracellular cytokine assay confirmed the findings that LCMV infection led to the generation of virus-specific CD8 T cells that cross-react with alloantigens, and further showed that virus-immune mice were refractory to the induction of tolerance by costimulation blockade [57]. Others have also reported that virus-immune mice are refractory to tolerance induction by costimulation blockade [53]. Because memory T cells are resistant to the induction of tolerance by costimulation blockade [107, 108], our data suggest that the allo-cross-reactive virus-specific memory T cells may precipitate the rejection of allografts even in the presence of costimulation blockade.

## 8. VIRUS INFECTION AND ESTABLISHED ALLOGRAFT SURVIVAL

Surprisingly, mice infected with LCMV one day after transplantation also exhibit shortened allograft survival [57]. Interestingly, the longer time after transplantation is, the less impact LCMV infection has on allograft survival. The deletion of alloreactive CD8^+^ T cells is thought to be complete at this time [41, 42], making it improbable that LCMV is interfering with deletion. However, because costimulation blockade protocols are only implemented during the peritransplant period, it is possible that LCMV infection shortly after transplantation prevents the generation of regulatory T cells, which have been shown to require up to 30 days after costimulation blockade to develop [129]. Further research is necessary to elucidate the mechanisms by which LCMV shortens allograft survival during the post-transplantation timeframe.

## 9. SUMMARY

Viral infection presents a potent barrier to the induction of transplantation tolerance. In this review, we have discussed potential mechanisms by which viral infection modulates organ allograft survival in the setting of transplantation tolerance. We have briefly summarized data on three mechanisms by which viral infection may mediate these effects: bystander activation, modulation of Tregs, or heterologous immunity. Recognition of viruses by pattern recognition receptors on innate immune cells can also directly stimulate the maturation of APCs, and thus may lead to bystander activation and licensing of alloreactive T cells. Activation of APCs by viruses may trigger the release of cytokines such as IL-6 that can prevent the generation and/or function of regulatory T cells that are essential for transplantation tolerance. Finally, heterologous immunity may be responsible for the discrepancy that has been encountered when tolerance strategies that work in specific pathogen-free rodent models fail when translated to nonhuman primates and to humans [130], which have been exposed to a variety of pathogens and thus have large memory T cell pools. Understanding the cellular and molecular mechanisms by which viruses and other microbial organisms modulate transplantation tolerance may lead to novel approaches that improve the efficacy of allogeneic organ transplantation.

## ABBREVIATIONS

APC: Antigen presenting cell
BMT: Bone marrow transplantation
CARD: Caspase recruitment domain
CARDIF: CARD adaptor inducing IFN-B
cDC: Conventional dendritic cell
CTL: Cytotoxic T lymphocytes
DC: Dendritic cell
DAI: DNA-dependent activator of IFN-regulatory factors
dsRNA: Double stranded RNA
DST: Donor-specific transfusion
GP: Glycoprotein
GVHD: Graft-versus-host disease
IFN: Interferon
IKK: I*κ*B kinase
IKK-I: Inducible I*κ*B kinase
IPS-1: Interferon-*β* promoter stimulator
IRAK: IL-1 receptor-associated kinase
IRF: Interferon regulatory factory
ISRE: Interferon-stimulated response element
LCMV: Lymphocytic choriomeningitis virus
mAb: Monoclonal antibody
MAVS: Mitochondrial antiviral signaling protein
MDA5: Melanoma differentiation factor-5
MCMV: Murine cytomegalovirus
MyD88: Myeloid differentiation primary response gene-88
NP: Nucleoprotein
OPN: Osteopontin
PRR: Pattern recognition receptor
PV: Pichinde virus
RIG-I: Retinoic acid inducible gene I
RLR: RIG-I-like receptor
pDC: Plasmacytoid dendritic cell
TBK-1: TANK-binding kinase 1
TCR: T cell receptor
TIR: Toll/interleukin-1 receptor
TLR: Toll-line receptor
TRAF: Tumor necrosis factor receptor-associated factor
Treg: Regulatory T cell
TRIF: TIR-domain-containing adaptor protein inducing IFN-*β*
VISA: Virus-induced signaling adaptor
VV: Vaccinia virus
WBI: Whole-body irradiation
